# Exploring In Vitro Biological Cellular Responses of Pegylated β-Cyclodextrins

**DOI:** 10.3390/molecules27093026

**Published:** 2022-05-08

**Authors:** Juliana Rincón-López, Miguelina Martínez-Aguilera, Patricia Guadarrama, Karla Juarez-Moreno, Yareli Rojas-Aguirre

**Affiliations:** 1 Laboratorio de Materiales Supramoleculares (SupraMatLab), Instituto de Investigaciones en Materiales, Universidad Nacional Autónoma de México, Circuito Exterior S/N, Ciudad Universitaria, Coyoacán 04510, Mexico; juliana.rincon@comunidad.unam.mx (J.R.-L.); miguelinamtz91@gmail.com (M.M.-A.); patriciagua@materiales.unam.mx (P.G.); 2 Centro de Física Aplicada y Tecnología Avanzada, Universidad Nacional Autónoma de México, (CFATA-UNAM), Blvd. Juriquilla #3001 Col. Jurica La Mesa CP, Querétaro 76230, Mexico

**Keywords:** β-cyclodextrin, polyethylene glycol, macrophages, osteoblasts, MDCK cells, cell viability, ROS, cell cycle, cell migration

## Abstract

βCDPEG5 and βCDPEG2 are two derivatives comprising seven PEG linear chains of 5 and 2 kDa, respectively, conjugated to βCD. As βCDPEGs display different physicochemical properties than their precursors, they could also trigger distinct cellular responses. To investigate the biological behavior of βCDPEGs in comparison to their parent compounds, we performed broad toxicological assays on RAW 264.7 macrophages, MC3T3-E1 osteoblasts, and MDCK cells. By analyzing ROS and NO_2_^−^ overproduction in macrophages, we found that βCDPEGs induced a moderate stress response without affecting cell viability. Although MC3T3-E1 osteoblasts were more sensitive than MDCK cells to βCDPEGs and the parent compounds, a similar pattern was observed: the effect of βCDPEG5 on cell viability and cell cycle progression was larger than that of βCDPEG2; PEG2 affected cell viability and cell cycle more than βCDPEG2; cell post-treatment recovery was favorable in all cases, and the compounds had similar behaviors regarding ROS generation. The effect on MDCK cell migration followed a similar pattern. In contrast, for osteoblasts, the interference of βCDPEG5 with cell migration was smaller than that of βCDPEG2; likewise, the effect of PEG2 was shorter than its conjugate. Overall, the covalent conjugation of βCD and PEGs, particularly to yield βCDPEG2, improved the biocompatibility profile, evidencing that a favorable biological response can be tuned through a thoughtful combination of materials. Moreover, this is the first time that an in vitro evaluation of βCD and PEG has been presented for MC3T3-E1 and MDCK cells, thus providing valuable knowledge for designing biocompatible nanomaterials constructed from βCD and PEGs.

## 1. Introduction

βCD, a cyclic oligomer bearing seven glucopyranose units linked by α-1,4 glycosidic bonds, is widely recognized to form inclusion complexes (ICs) through host/guest interactions with low polarity molecules. The chemical versatility of βCD, enabling its random or selective functionalization, has resulted in numerous βCD derivatives displaying various physicochemical and biological features [1]. Some of these derivatives (i.e., HPβCD and SBEβCD) are used in the pharmaceutical field to form ICs with drugs to enhance their aqueous solubility and stability. Nonetheless, novel applications, related to or based on the solubility improvement, reveal the potential of βCDs derivatives to develop practically any pharmaceutical technology [2,3].

The chemical derivatization of βCD also includes obtaining polymeric and amphiphilic βCD-based materials suitable to build functional molecular nanoplatforms envisaging controlled drug release. Most research on these platforms centers on nanomaterial engineering (size, shape, functionalities), drug loading, and biological evaluations generally performed in specific types of cells only to assess the applications for which the nanomaterial was designed. It is known that cells respond differently to the nanomaterial size and shape, and even a subtle change in the nanomaterial chemical composition may elicit a different biological response [4,5]. Therefore, systematic in-depth biological evaluations of drug-free βCD-based nanomaterials and their components, at their early stages of development, are fundamental to better understand their effects at cellular and subcellular levels to make them succeed in their journey to actual biomedical applications.

**Figure 1:**
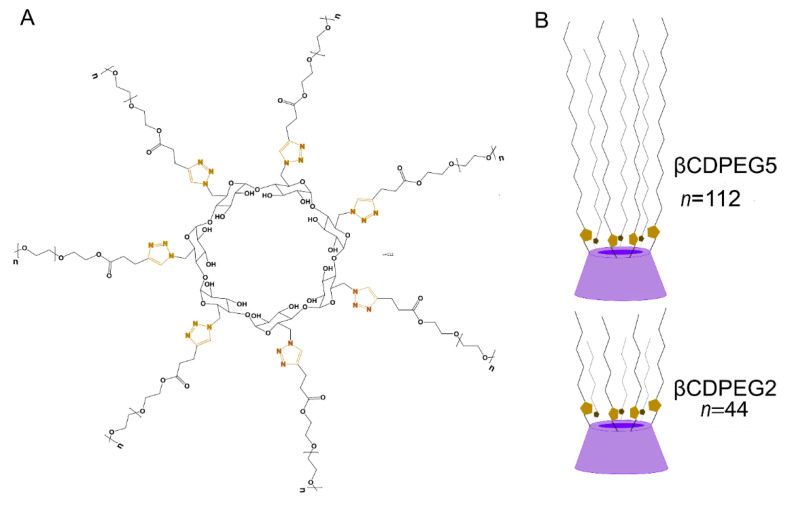
(**A**) Chemical structure and (**B**) schematic representation of βCDPEG5 and βCDPEG2.

We previously synthesized βCDPEG2 and βCDPEG5, two βCD derivatives obtained through the selective conjugation of seven PEG chains of 2 and 5 kDa, respectively, to the βCD primary face (Figure 1) [6]. 

Depending on their concentration in aqueous media, βCDPEG5 molecules can be as individual entities, as dimers, or can self-associate to yield stable spheric nanoparticles of ~150 nm with a cac of 0.5 mM [7]. A similar behavior has been observed for βCDPEG2 (data not shown). This attractive feature of βCDPEGs, which are devised as drug delivery systems, would allow selecting the most convenient configuration to carry drugs (as inclusion complexes, encapsulated in the nanoparticle, or both). Although βCD and PEG are considered safe components, βCDPEGs are new chemical entities that could interact at the biological interfaces differently from their parent compounds. Therefore, we performed preliminary in vitro viability studies in HeLa and Vero cells and human monocytes, finding that βCDPEGs had a null effect on the viability of those cell lines in the range of 25–500 µg/mL [6]. 

To continue investigating the biological behavior of βCDPEGs and envisioning the creation of a “biological library” of these materials for their use in the rational design of drug delivery systems, in this work, we explored specific in vitro cellular responses to βCDPEG5 and βCDPEG2 in three different in vitro animal cell models: RAW 264.7 macrophages, MC3T3-E1 osteoblasts, and Madin–Darby canine kidney (MDCK) cells. The experiments were performed at concentrations ranging from 25 to 500 µg/mL (0.00067–0.0135 mM), which were below the cac (0.5 mM). Therefore, the biological behavior presented herein corresponded to that of βCDPEG individual entities only. 

Macrophages play an essential role in the regulation of inflammation and immune response and also in removing nanomaterials through phagocytosis from the bloodstream before reaching the therapeutic target. Pegylation is still the most used strategy to decrease the phagocytic cell uptake, thus prolonging the systemic lifetime of nanomaterials. Recent investigations have shown that pegylation could not reduce phagocytic uptake [8] and could even increase the internalization of nanomaterials by cells such as human neutrophils [9]. The varied evidence of the PEG stealth capacity has been attributed to PEG MW, architecture, and density [9,10,11,12]. Therefore, investigating the interactions between macrophages and pegylated materials at their early stages of development must be imperative. Although RAW264.7 has biological features distinct from human peripheral blood-derived macrophages, these cells comprise a standard model to evaluate in vitro immune activity, inflammation, and certainly, the biological effect of pegylated nanomaterials [13,14]. 

The investigation of synthetic materials for bone tissue regeneration is markedly growing. In this regard, the MC3T3-E1 osteoblast cell line has been a convenient in vitro model to study biocompatibility and molecular mechanisms for the development of osteoblasts [15]. Envisioning βCDPEG applications beyond drug carriage and, given the potential of βCD-materials for implants, scaffolds, and bone engineering applications [3,16,17], we were motivated to explore the effect of βCDPEGs on the MC3T3-E1 cell line. 

MDCK cells are used to evaluate various biological aspects, including drug transport, permeability, and nephrotoxicity. In this work, we used them as a model for epithelial cells [18,19,20].

For RAW 264.7 macrophages, we assessed the effect of the pegylated compounds on their viability and the generation of radical species (ROS and NO). For MC3T3-E1 and MDCK cells, we evaluated the effect of βCDPEGs on their viability, cell cycle progression, and ROS production; we also performed permeabilization assays and explored the βCDPEGs cytostatic effect through cell migration experiments.

In all of the studies, we included the precursors, βCD, and PEGs to compare the elicited biological responses between them and their conjugates, intending to infer the structure–biological response relationships of βCDPEGs. 

Moreover, although βCD and its derivatives have been considered safe excipients for specific pharmaceutical formulations, their novel uses are being deployed (active pharmaceutical ingredients, vaccine adjuvants, and excipients used in alternative administration routes) [2,3]. Thus, an in-depth biological investigation of βCD and its derivatives is still highly relevant [21,22]. On the other hand, it has been recently reported that PEG, considered biologically inert, can trigger immune responses and elicit cytotoxicity [23,24]. Hence, despite the overwhelming enthusiasm for the pegylation approach, an adequate assessment of its biological performance is required for its actual success. Essentially, this is the first time that a broad in vitro evaluation of βCD and PEG has been presented in MC3T3-E1 and MDCK cell line models, providing valuable knowledge for the community devoted to designing βCD-based materials and pegylated systems. 

## 2. Results and Discussion

### 2.1. RAW 264.7 Macrophages

The interaction between macrophages and pegylated nanomaterials is crucial as it impacts the systemic behavior of those materials, as well as their toxicological profile. In the attempt to inform the preliminary effects of βCDPEGs, we investigated their effect on the macrophages’ viability and on the overproduction of radical species (ROS and NO).

#### 2.1.1. Cell Viability

**Figure 2:**
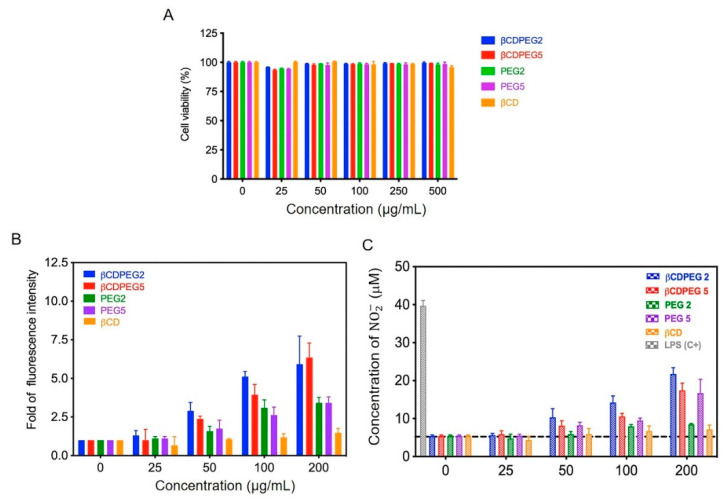
Effect of βCDPEGs, βCD, and PEGs on (**A**) cell viability, (**B**) ROS, and (**C**) NO_2_^−^ production in RAW264.7 macrophages. Dashed line indicates the endogenous concentration of NO_2_^−^ in macrophages grown without treatments. LPS: Lipopolysaccharides used as positive control (C+).

Figure 2A shows that βCDPEGs did not affect the viability of RAW 264.7. This was also the case for βCD, whose behavior was consistent with that in other works in which CDs (αCD, βCD, MβCD, and HPβCD) did not alter macrophage viability in concentrations ranging from 0.001 to 1 mM [25,26,27,28,29,30]. The viability of RAW 264.7 cells was not affected by PEGs either. 

#### 2.1.2. ROS and Nitrite (NO_2_^−^) Generation

ROS production in macrophages is triggered by phagocytosis and diverse endogenous (i.e., cytokines) and exogenous (i.e., chemicals) signals. Although ROS can modulate cellular functions and macrophage-mediated immunity, their overproduction induces oxidative stress, which could cause damage to cellular proteins, lipids, and DNA [31,32]. Nitrites (NO_2_^−^) form by the oxidation of nitric oxide (NO), which is one of the pro-inflammatory mediators secreted by macrophages to activate both immune response to pathogens and oxidative and inflammation processes [33]. 

In this work, we evaluated the effect of βCDPEGs, βCD, and PEGs on ROS overproduction. We also quantified NO_2_^−^ as an indirect approach to determine NO generation. 

Figure 2B shows that βCDPEG5 and βCDPEG2 elevated intracellular ROS levels in a dose-dependent manner, reaching a maximum fluorescence, at 200 µg/mL, of ~6.1-fold higher than in the untreated cells. Free PEGs produced lower levels of ROS, as only an increase of ~3.3-fold in the fluorescence, compared to the control, was observed at 200 µg/mL, which was half the effect of the βCDPEG conjugates. The difference in ROS levels between βCDPEGs and PEGs may be attributed to PEG architecture (star polymer vs. linear PEG) and density (seven grouped PEG chains vs. free linear PEGs). 

βCDPEG5, βCDPEG2, and PEG5 elevated ~2.9-fold the concentration of NO_2_^−^ at 200 µg/mL in comparison to the negative control. There were no significant effects in NO_2_^−^ levels exerted by PEG2. In this case, rather than PEG architecture, it seems that MW was the factor that most influenced the NO_2_^−^ production in RAW 264.7 cells (Figure 2C). 

The responses of RAW 264.4 to βCD observed in Figure 2B,C are concordant with Davaatseren 2017, whose report indicated that βCD does not affect RAW 264.7 viability or ROS and NO production [29].

Basically, βCDPEGs moderately augmented the production of ROS and NO_2_^−^ to a greater extent than the parent compounds, without compromising macrophages’ viability. Although other studies such as those on cellular uptake will give deeper insight into the macrophages’ response to βCDPEGs, our preliminary results provided relevant information about the relationship between pegylated structures and macrophage responses that can contribute to those devoted to the design of pegylated materials.

### 2.2. MC3T3-E1 Osteoblastic Cell Line

CDs are proving to be attractive for bone engineering applications. On the one hand, they can form ICs with osteoinductive drugs such as simvastatin and melatonin to enhance their aqueous solubility, thus improving their osteogenic differentiation efficiency [16,34]. On the other, CDs can be used to construct polymeric or hybrid drug-loaded scaffolds, implants, and coatings for osteoinductive and anticarcinogenic performance, in which the use of CDs optimizes the bioactivity of therapeutic molecules [35,36,37,38,39,40,41,42] and even the systems’ rheological properties [43]. Either way, any CD-based material considered for bone engineering must be biocompatible with bone cells. Thus, we were interested in studying the effect of our pegylated CDs in the MC3T3-E1 cell line.

#### 2.2.1. Cell Viability

**Figure 3:**
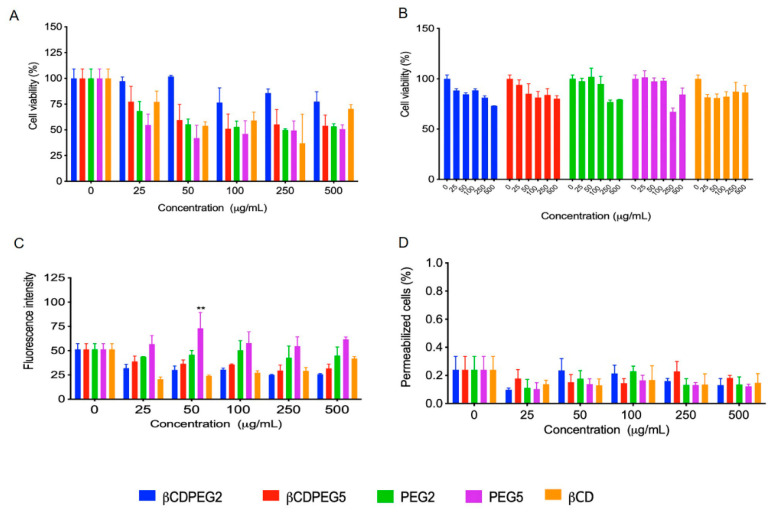
Effect of βCDPEGs, βCD, and PEGs on MC3T3-E1 osteoblast (**A**) cell viability; (**B**) cell viability recovery assay; (**C**) ROS overproduction; and (**D**) cell membrane permeability. All results are expressed as the mean ± SD (*n* = 3), ** *p* < 0.01 using two-way ANOVA with Dunnett’s multiple comparison tests.

Figure 3A shows that βCDPEG2 was not cytotoxic to MC3T3-E1 cells from 25 to 50 µg/mL, whereas cell viability was around 80% in the 100–500 µg/mL concentration range. The effect of free PEG2 on osteoblasts was more significant than that of βCDPEG2: at the initial concentration of 25 µg/mL, cell viability was 68% and then decreased to 55% at 50 µg/mL and persisted close to this value for the rest of the concentrations. Surprisingly, it seems that the star-shaped architecture and higher density of PEG in the βCDPEG2 macromolecule decrease its cytotoxicity.

A concentration-dependent effect was observed for βCDPEG5 that ranged from 23% of cell viability, at the lowest concentration, to 55% at 50–500 µg/mL. PEG5 also affected the viability of MC3T3-E1 cells more than its conjugate, βCDPEG5, but this occurred only at 25 and 50 µg/mL. At higher concentrations, the cytotoxicity of both PEG5 and βCDPEG5 was similar. In this case, the structural features of PEG were not correlated to the biological response.

Pegylated (PEG MW in the range of 5 to 20 kDa) scaffolds and platforms for delivering bioactive molecules to promote osteogenic differentiation have been evaluated in MC3T3-E1 cells [44,45,46]. The pegylated materials did not seem to hamper cell viability; however, it is worth noting that the effect of free PEG is not generally studied. Nevertheless, it must be underscored that the effect of free PEG should not be disregarded as the polymer might impact specific biological responses, as we have shown herein. 

Previous investigations of the effect of βCD ICs or βCD-based bioactive platforms on MC3T3-E1 cell viability have not included the biological effect of βCD itself [16]. In this work, we have shown for the first time that βCD fairly affects cell viability at the evaluated concentration range. 

To sum up, the effects on cell viability from βCD and PEG2 were mitigated when conjugated as βCDPEG2; the behavior of βCD, PEG5, and βCDPEG5 was pretty similar; the effect of βCDPEG5 on MC3T3-E1 osteoblast viability was higher than that of βCDPEG2, and overall, our results suggest that MC3T3-E1 osteoblasts respond to PEG MW changes.

#### 2.2.2. Post-Treatment Recovery Assay

A recovery assay was performed to evaluate whether βCDPEGs, βCD, and PEGs could induce a cytostatic effect. For this purpose, cells were washed out to remove the compounds and then cultured again to determine their capacity to proliferate through the analysis of the viable cells after 24 h, as shown in Figure 3B. Strikingly, the viability of MC3T3-E1 cells was above 80% for all of the compounds in the entire concentration range, meaning that despite the reduction in their viability, cells can completely recover once the pegylated compounds are removed.

#### 2.2.3. ROS Generation

Oxidative stress arising from excessive levels of ROS is a major cause of bone diseases, including osteoporosis [47]. Therefore, we were interested in evaluating the generation of ROS in MC3T3-E1 as a possible cell death mechanism. 

Figure 3C shows that βCDPEG conjugates, βCD, and PEG2 did not induce overproduction of ROS. However, PEG5 slightly increased ROS levels and behaved similarly at all concentrations. Our results suggest that PEG MW influenced ROS generation in MC3T3-E1 cells. This particular finding coincides with a previous work reporting ROS overproduction as the mechanism of cytotoxicity of PEG derivatives (MWs ranging from 400 to 4000 Da) on L929 cells [25] To note, the conjugation of PEG5 and βCD, in the form of βCDPEG5, limited ROS overproduction. 

#### 2.2.4. Membrane Permeability

Considering that some βCD derivatives increase membrane permeability [48], we also performed permeabilization assays. The influence of βCDs on cell membrane permeability depends on the hydrophobic degree of the βCD derivative, the cavity size, and the type of cell. Figure 3D shows that βCD and its pegylated derivatives did not induce any change in the permeability of MC3T3-E1 cell membranes. The same results were observed for free PEGs.

#### 2.2.5. Cell Cycle

The effect of PEG and βCD on cell cycle progression has been scarcely explored. Parnaud et al. reported that PEG (7.5–10 mM, MW 8 kDa) induced cell cycle arrest in the G0/G1 phase of HT29 cells [49]; a similar effect was observed for MβCD in the cell cycle of RAW 264.7 macrophages [50]. Therefore, investigating the role of free PEG and βCDs and their combinations, such as βCDPEGs, in cell cycle progression provides valuable information for their actual use as drug delivery systems.

**Figure 4:**
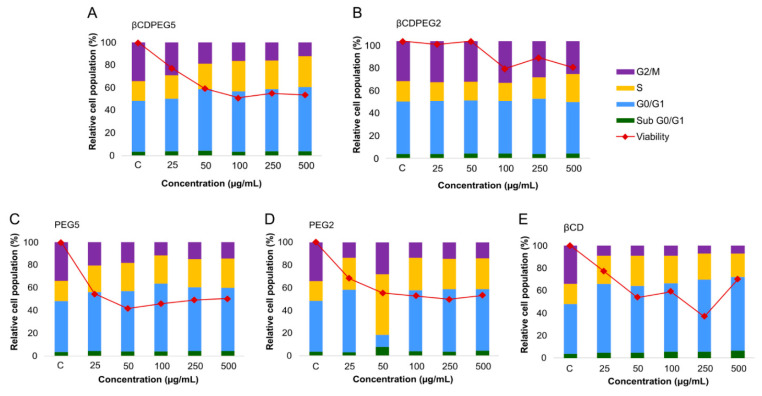
Effects of (**A**) βCDPEG5, (**B**) βCDPEG2, (**C**) PEG5, (**D**)PEG2, and (**E**) βCD on the cell cycle of MC3T3-E1 osteoblasts. Viability refers to the % cell viability reported in Section 2.2.1, which was included to facilitate comparison with % relative cell population.

Figure 4 summarizes the comparison between the effects of βCDPEGs, βCD, and PEGs on the cell cycle of MC3T3-E1 osteoblasts (Table S1 in Supplementary Material shows the % of relative cell population for each cell cycle stage). As observed, there were no changes in the cell cycle progression of osteoblasts after exposure to βCDPEG2, except at the concentration of 500 µg/mL, where a faint disruption in the percentage of cells in the G2/M phase was observed in comparison to the untreated cells. PEG2, in the whole range, caused a mild increase in the number of cells in the S phase, with a subsequent decrease in the G2/M. The effect of PEG2 was more significant than that of βCDPEG2, evidencing the advantageous covalent conjugation of βCD and PEG2. βCDPEG5 at concentrations from 50 to 500 µg/mL caused an increase in the percentage of cells in the G0/G1 and S phases while decreasing the osteoblast population in G2/M. Similar outcomes were attained for its counterpart, PEG5. Results show that PEG MW impacted the osteoblasts’ cell cycle progression, and this effect was independent of PEG density, the covalent conjugation, and the molecule architecture. PEG5 and βCDPEG5 interfered at the G1 and S levels, indicating that osteoblasts were not ready to initiate DNA replication due to possible damage, thus preventing cells from reaching the mitotic stage [51]. For βCD, at concentrations higher than 100 µg/mL, only the G0/G1 phase was arrested. These outcomes were assuredly concordant with the decrease in cell viability (Section 2.2.1) after incubation with PEG5 and βCDPEG5. Additional studies to determine whether DNA damage is the cause of cell cycle arrest would enormously contribute to base structure–property relationships.

#### 2.2.6. Cell Migration

**Figure 5:**
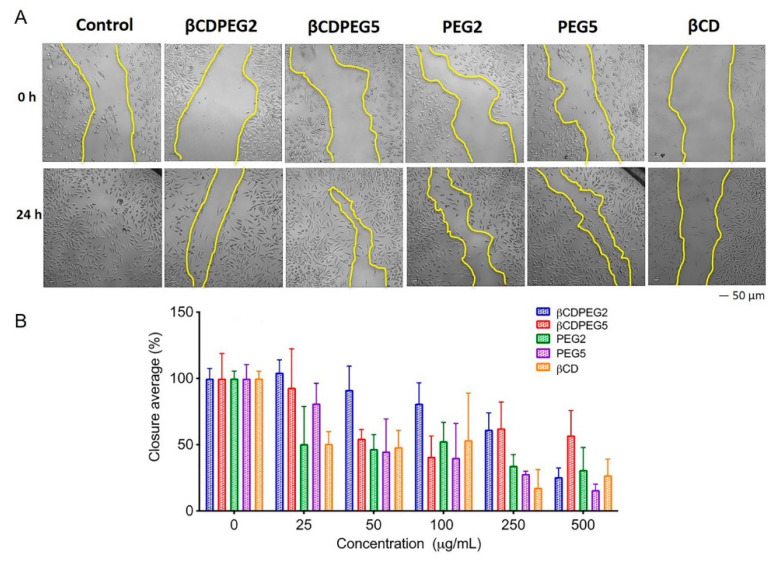
MC3T3-E1 osteoblast migration in the presence of βCDPEGs, βCD, and PEGs. A scratch was made through the MC3T3-E1 cell layer, and then cells were cultured in the presence of different concentrations of βCDPEGs, βCD, and PEGs (25, 50, 100, 250 and 500 μg/mL) for 24 h. (**A**) Representative images of migration assay of the compounds at 500 μg/mL after the scratch (0 h) and at 24 h later, showing the gap closure (area between the two yellow lines). The images were captured at the same scale with a scale bar of 50 μm. (**B**) Area between the two dotted lines expressed as the percentage of cell closure relative to the control.

Cell migration studies, also known as scratch assays, assess the motility of cells through their ability to migrate and close a wound made in a confluent cell monolayer. Figure 5 shows that cell migration remained unchanged in the presence of βCDPEG2 at 25–100 µg/mL. However, from 250 to 500 µg/mL, cell migration was significantly inhibited in a dose-dependent manner, with a gap closure of 26% at the highest concentration. Its PEG2 counterpart hampered cell migration at all concentrations, holding the gap closure at around 50% in all cases. Above 25 µg/mL of βCDPEG5, wound closures were above 50%, while the effect of free PEG5 on cell migration was considerably higher.

In this case, a different pattern was observed as the effect of βCDPEG2 was more extensive than that of βCDPEG5. Among all of the evaluated molecules, PEG5 affected MC3T3-E1 osteoblast migration the most, but its effect was lessened when conjugated with βCD.

It is worth noting that βCD and its derivatives can disturb cell migration, as shown by Maki et al. 2020 with βCD in Caco-2 cells or Guerra et al. 2016 with MβCD in MDA-MB 231 cells [52,53]. We also observed that βCD shortened MC3T3-E1 osteoblast migration in the present work. At 25–100 µg/mL, the closure gap was close to 50%, whereas at 250–500 µg/mL, it remained around 25%. These effects could be attributed to the well-known ability of CDs to form ICs with cholesterol cell membranes; however, in-depth studies to confirm this statement are required.

Unexpectedly, the effect of PEG2 on cell migration was shorter than that of βCDPEG2. Under the same experimental conditions, more studies evaluating a series of different MW would be required to identify PEG structure–cell migration relationships. On the other hand, deeper investigations, for example, in the mechanobiology field, would allow us to integrate physical and molecular insights on cell migration and cell cycle arrest in the presence of βCDPEGs.

βCDPEGs, βCD, and PEGs decreased MC3T3-E1 cell viability and restrained cell growth. However, since our results did not show disruption of membrane integrity or ROS overproduction, the effects could be due to cell motility inhibition rather than a cytotoxic activity. This postulate is supported by the observations in cell migration and post-treatment viability. 

We have informed the effect of βCDPEGs on MC3T3-E1 osteoblasts at different levels. Moreover, MC3T3-E1 cells’ response in the presence of free PEGs and native βCDs was herein presented for the first time. Overall, we have shown that the biocompatibility profile of βCD and PEG is optimized when conjugated in the form of βCDPEGs. So far, it would be plausible to suggest that at concentrations above 250 µg/mL, βCDPEG2 could exert a synergic effect with cytotoxic drugs to treat bone malignancies. On the other hand, βCDPEG5 could be used to develop novel biomaterials for bone regeneration and tissue engineering applications.

### 2.3. MDCK Cells

#### 2.3.1. Cell Viability

**Figure 6:**
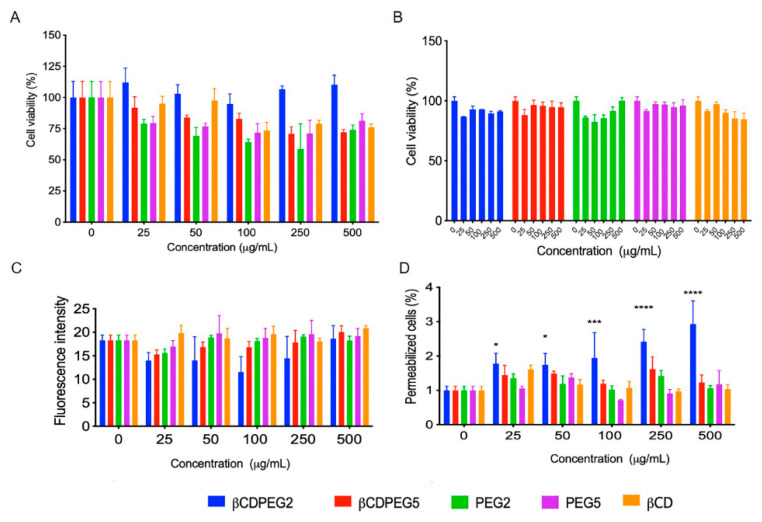
Effects of βCDPEGs, βCD, and PEGs on MDCK cells. (**A**) Cell viability; (**B**) recovery of MDCK cells; (**C**) ROS overproduction; and (**D**) cell membrane permeability. All results are expressed as the mean ± SD (*n* = 3) * *p* < 0.05; *** *p* < 0.001; **** *p* < 0.0001 using two-way ANOVA with a Dunnett’s multiple comparison tests.

We analyzed the effects of βCDPEG materials and their single components on MDCK cells. As observed in Figure 6A, βCDPEG2 did not alter cell viability, while in response to βCDPEG5, free PEGs, and βCD, it remained close to 80%.

As we have mentioned throughout this document, PEG is considered a biologically inert polymer. Nonetheless, it could elicit a biological response depending on its MW, functional terminal groups, concentration, and architecture [54]. Herein, we detected that PEGs moderately affected MDCK cells’ viability, around 75%, regardless of their MW. There was no difference between PEG5 and the conjugate βCDPEG5. To our knowledge, this is the first report on the effect of free PEG2 and PEG5 on the viability of MDCK cells.

The response of MDCK cells to βCD observed herein was concordant with previous works: Francis et al. estimated 95% of cell viability in the presence of MβCD 10 Mm (14,295 µg/mL), similarly to Hailstones et al., when they explored MDCK viability exposed to trimethyl-βCD at 1000 µg/mL [55,56]. Pentacyclic triterpene-functionalized βCDs, designed to inhibit the activity of the influenza virus, did not exert cytotoxicity against MDCK cells at ~500 µg/mL [57,58].

#### 2.3.2. Post-Treatment Recovery Assay

Once βCDPEG5, PEGs, and βCD were removed from the cell media, cell viability was recovered up to 90%, and the evaluated compounds did not exert a cytostatic effect on MDCK cells (Figure 6B).

#### 2.3.3. ROS Generation

As depicted in Figure 6C, βCDPEGs did not induce ROS overproduction—even βCDPEG2, which did permeabilize the cell membrane (see below). Similar outcomes were observed for the parent compounds. As far as we know, this is the first time ROS generation on MDCK cells in response to βCDs has been investigated.

#### 2.3.4. Membrane Permeability

βCDPEG2 moderately altered MDCK cells’ membrane permeabilization in a concentration-dependent manner (Figure 6D). Wang et al. recently reported that free low MW PEGs (˃2 kDa) cross cell membranes of MDCK cells by passive diffusion. In contrast, PEGs between 5–20 kDa internalize by a combination of passive diffusion and caveolae-mediated endocytosis [59]. Therefore, additional studies will be valuable to know whether the change in membrane permeability results in βCDPEG2 internalization and why βCDPEG5 and free PEGs did not affect membrane permeabilization.

#### 2.3.5. Cell Cycle

**Figure 7:**
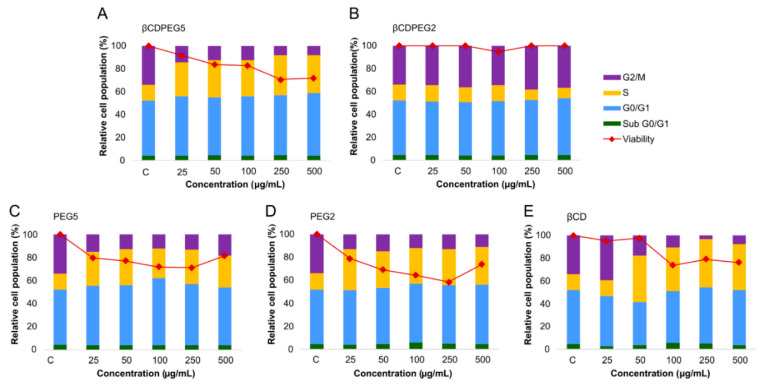
Effects of (**A**) βCDPEG5, (**B**) βCDPEG2, (**C**) PEG5, (**D**) PEG2, and (**E**) βCD on the cell cycle of MDCK cells. Viability refers to the % cell viability reported in Section 2.3.1, which has been included to facilitate comparison with the % relative cell population.

Figure 7 shows the effect of βCDPEGs, βCD, and PEGs on MDCK cells’ cycle progression (Table S2 in Supplementary Material displays the % of relative cell population for each cell cycle stage). βCDPEG2 did not alter the cell cycle, whereas PEG2 augmented the cell population at the S phase with a subsequent decrease in cells at G2/M. Likewise, βCDPEG5 and PEG5 arrested the cell cycle at the S phase. The response to βCDPEG5 was more significant than that to PEG5, evidencing, in this case, the influence of PEG density and architecture on cellular responses; the effects of PEG2 and PEG5 were similar. The arrest at the S phase might indicate that cells are responding to DNA damage triggered by the exposure to βCDPEGs, βCD, and PEGs and attempting to repair it if there is any [51]. βCD also arrested the cell cycle at the S phase. This effect was initiated at 50 µg/mL, unlike the pegylated compounds, whose effect was observed at 25 µg/mL.

Interestingly, the conjugate βCDPEG2 resulted in a favorable approach to clear away the cell cycle arrest induced by βCD and PEG2. The interference of the other compounds with the MDCK cells cycle could be correlated to the moderate decrease in cell viability. As occurred with osteoblasts, βCDPEG2 was the compound with fewer effects on cell cycle progression.

#### 2.3.6. Cell Migration

**Figure 8:**
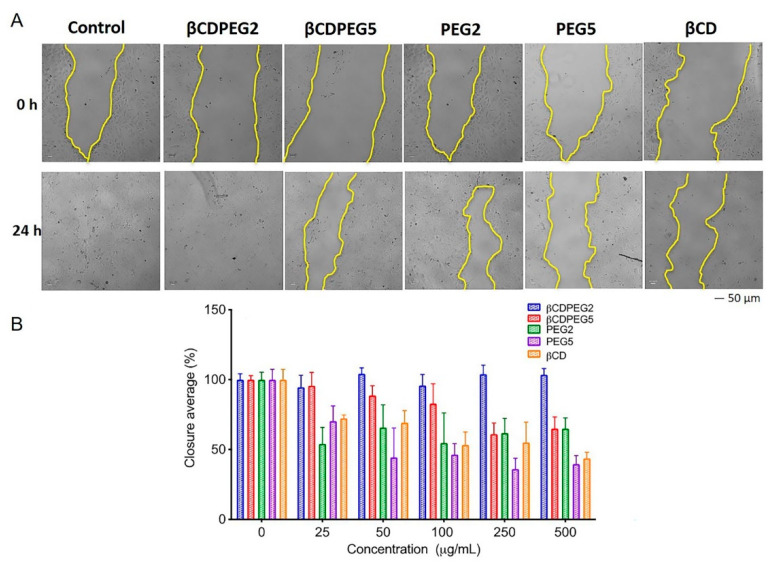
MDCK cell migration in the presence of βCDPEGs, βCD, and PEGs. A scratch was made through the MC3T3-E1 cell layer, and then cells were cultured in the presence of different concentrations of βCDPEGs, βCD, and PEGs (25, 50, 100, 250 and 500 μg/mL) for 24 h. (**A**) Representative images of migration assay of the compounds at 500 μg/mL after the scratch (0 h) and at 24 h later showing the gap closure (area between the two yellow lines); the images were captured at the same scale with a scale bar of 50 μm. (**B**) Area between the two dotted lines expressed as the percentage of cell closure relative to the control.

Figure 8 shows that the wound closure for MDCK cells exposed to βCDPEG2 was 100% at all evaluated concentrations. In contrast, the gap was around 60% in cells incubated with PEG2 in the entire concentration range. βCDPEG5 at 250–500 µg/mL kept the gap closure around 60%; lower concentrations did not interfere with cell migration. Its counterpart, PEG5, had a notable effect at 250–500 µg/mL, with a gap closure below 50%. In the range of 25–100 µg/mL, PEG5 mildly hindered cell migration. βCD also showed a decremental relationship between the gap closure and the concentration, going from 72% at 25 µg/mL to 44% at 500 µg/mL. As we mentioned in Section 2.2.6, previous studies have shown that βCD interferes with the migration of different cells. Hence, we provide cumulative evidence about the effect of βCD on cell motility, which encourages further studies to elucidate the cellular and molecular mechanisms involved.

Strikingly, in both cases, the conjugation between PEGs and βCD decreased the parent compounds’ effect on MDCK cells’ motility. In particular, βCDPEG2 promoted cell migration, a desirable attribute if we consider βCDPEG2 for its use as a scaffold for epithelial cells in the tissue engineering field. Although extensive studies for inflammation, cell proliferation, and adhesion are required, we are opening the door to the possible uses of the pegylated conjugate in the entire nanomedicine field.

MC3T3-E1 osteoblasts were more sensitive than MDCK cells to βCDPEGs. Although MDCK and MC3T3-E1 cells comprise distinct cellular models, it was possible to identify some patterns in the biological response to the evaluated compounds.

As observed in Table 1, which summarizes the biological behavior of free PEGs and βCDPEGs, the effect of βCDPEG5 on cell viability was more significant than that of βCDPEG2. Free PEG2 affected cell viability more than did βCDPEG2; PEG5 behaved similarly to βCDPEG5. Cell post-treatment recovery was favorable in all cases.

ROS production was moderately induced only by PEG5 in MC3T3-E1 cells. The other compounds did not significantly trigger ROS overproduction and showed comparable behavior in both cellular models.

The interference in cell cycle progression was more extensive in the presence of βCDPEG5 than with βCDPEG2. The cell cycle arrest after incubation with PEG2 was more substantial than with βCDPEG2.

MDCK cells migration studies displayed the same pattern: βCDPEG2 did not interfere with cell motility, as the closure average was 100% in all concentrations, unlike βCDPEG5, whose closure average decreased; the same was true for PEG2. Likewise, the gap produced by PEG5 was higher than that by βCDPEG5. Surprisingly, this was a different response than that observed in osteoblasts, in which βCDPEG5 could be used to develop novel biomaterials for bone regeneration and tissue engineering applications.

The extent of the effect of the evaluated compounds depends on the cell line; in turn, the cellular response depends on PEG MW and molecular architecture in some cases. The covalent conjugation of βCD and PEGs, particularly with PEG2, appears to be beneficial in terms of biocompatibility.

We have provided essential biological information to rationally guide the potential applications of βCDPEGs in the nanomedicine field.

## 3. Methodology

### 3.1. Materials

The βCDPEG molecules used in this work belong to the batches whose synthesis and characterization were previously reported by our research group [6].

### 3.2. Cell Culture

Macrophages RAW 264.7 (TIB-71), pre-osteoblasts MC3T3-E1 subclone 4 (CRL-2593), and kidney MDCK cells (CCL-34) were purchased from the American Type Culture Collection (ATCC). Pre-osteoblast MC3T3-E1 were cultured in alpha-Minimum Essential Medium Eagle Medium (alpha-MEM, Sigma-Aldrich, St. Louis, MO, USA), and both macrophage and kidney cells were propagated in Dulbecco’s Modified Eagle’s Medium (DMEM, Sigma-Aldrich, St. Louis, MO, USA). All culture media were supplemented with 10% fetal bovine serum (FBS, BenchMark, Gemini Bio Products, Sacramento, CA, USA), 1% penicillin streptomycin (Sigma-Aldrich, St. Louis, MO, USA), 1% L-glutamine (BenchMark Gemini Bio Products, Sacramento, CA, USA), and 1.5 g/L sodium bicarbonate (Sigma-Aldrich St. Louis, MO, USA), and incubated until confluence at 37 °C in a 5% CO_2_ atmosphere.

### 3.3. Instrumentation

Flow cytometry was used to measure ROS generation, cell membrane permeability and cell cycle progression upon exposure of cells to different concentrations of βCDPEGs, βCD, and PEGs. The data were acquired using an Attune NxT flow cytometer equipped with blue and violet lasers (Life Technologies, Carlsbad, CA, USA). Unless specified, data consisted of 10,000 events (cells) computed in triplicate in three independent experiments for each sample. The Attune NxT acquisition software version 3.2.1. (ThermoFisher, Applied biosystems, Waltham, MA, USA) was used for data analysis.

### 3.4. Cell Viability Assay

The susceptibility of RAW 264.7, MC3T3-E1, and MDCK cells to βCDPEGs, βCD, and PEGs was evaluated in a 96-well plate with 10,000 cells per well. The viability of cells was tested by reducing MTT reagent 3-(4,5-dimethylthiazol-2-yl)-2,5-diphenyltetrazolium (Sigma-Aldrich, St. Louis, MO, USA) following the instructions of the manufacturer, as follows. Cells were seeded for 24 h in cell culture media at 37 °C in a 5% CO_2_ atmosphere. Then, cell medium was discarded, and different amounts of the compounds (from 25 to 500 µg/mL) were added to a final volume of 100 µL of cell medium. Then, cells were incubated for 24 h at 37 °C and 5% CO_2_. After this, cell culture medium was removed, cells were rinsed thrice with 200 µL of PBS 1x, and MTT cytotoxic determination assay was carried out. Untreated cells were used as control for cell viability, while negative control was assessed using 100 µL of 0.5% Triton X-100 in PBS. Absorbance measurement of MTT reduction was recorded with a 96-well plate reader (GoScan, Thermo Scientific, Waltham, MA, USA). Background absorbance of cell viability was measured at 690 nm and subtracted from the absorbance values of MTT reduction due to cell viability recorded at 570 nm. Experiments were performed independently in a threefold manner with internal triplicates.

### 3.5. Production of Nitrites by Macrophages

Griess assay was used to measure the production of nitrites by macrophages upon incubation with βCDPEGs, βCD, and PEGs. Macrophages were seeded in a 96-well plate at a density of 10,000 cells per well and incubated for 24 h at 37 °C and 5% CO_2_. After this, different concentrations, from 25 to 500 µg/mL, of βCDPEGs, βCD, and PEGs were added to the wells and incubated for 24 h at 37 °C and 5% CO_2_. Then, 20 µL of cell media from each well were mixed in a new well with 80 µL of 5 mM sodium nitroprusside (Sigma-Aldrich, St. Louis, MO, USA) and incubated for 1 h in darkness at 37 °C and 5% CO_2_. Then, the reaction was mixed with 100 µL of Griess reagent solution (0.1% sulfanilamide and 0.1% N-(1-naphthyl ethylenediamine) (Sigma-Aldrich, St. Louis, MO, USA) and incubated at 25 °C for 15 min in darkness. The absorbance of the samples was read at 540 nm, and the values were compared with a standard curve using 1.67–100 µM of sodium nitrite as a reference reagent of nitrite production. Three independent experiments were performed with internal triplicates. Lipopolysaccharides (LPS) were used as positive control (C+) in a concentration of 100 ng/mL.

### 3.6. Reactive Oxygen Species Production

ROS production was measured by flow cytometry. Briefly, 50,000 RAW 264.7, MC3T3-E1 or MDCK cells were seeded in a 24-well plate and incubated for 24 h with βCDPEGs, βCD, and PEGs in concentrations from 25 to 500 µg/mL at 37 °C and 5% CO_2_. Then, cells were rinsed with PBS 1X and incubated with 30 μM of 2’,7′-dichlorofluorescein diacetate for 90 min at 37 °C and 5% CO_2_. After this, cells were rinsed, harvested, and resuspended in PBS 1X for flow cytometry measurements, using the BL-1 channel for a 488 nm excitation and 525 nm emission lasers. The endogenous and basal level of ROS for each cell line was recorded on cells without βCDPEGs, βCD, or PEGs treatments.

### 3.7. Post-Treatment Recovery Assay (Re-Cultivation Assay)

To determine whether exposing MC3T3-E1 or MDCK cells to βCDPEGs, βCD and PEGs induced a cytostatic effect, we carried out a post-treatment recovery assay, also named re-cultivation. Briefly, after incubating cells with βCDPEGs, βCD, and PEGs, cells were rinsed, harvested, and placed in a new 96-well culture plate and incubated at 37 °C in a 5% CO_2_ atmosphere with cell culture medium without treatment for 24 h. After that, we analyzed cells by the same procedure as used in the MTT cell viability assay.

### 3.8. Cell Membrane Permeability Assay

Upon incubation of MC3T3-E1 or MDCK cells in the presence of different concentrations of βCDPEGs, βCD, and PEGs, cells were harvested by trypsinization and resuspended in 200 µL of PBS 1X and then incubated for 30 min with propidium iodide (Sigma-Aldrich, St. Louis, MO, USA) (PI) at 25 °C to measure the permeability of the cell membrane. Afterward, cells were rinsed three times with PBS and further analyzed by flow cytometry using the BL-3 channel for PI detection.

### 3.9. Cell Cycle Progression Measurements by Flow Cytometry

Cells at a density of 10,000 cells per well were seeded in a 96-well culture plate and incubated with different concentrations of βCDPEGs, βCD, and PEGs, for 24 h at 37 °C in a 5% CO_2_ atmosphere. After this, cells were harvested by trypsinization and resuspended with 1 mL of 70% ice-cold ethanol. Cells were fixed by incubation at 4 °C for 1 h. Then, cells were centrifuged and resuspended in 1 mL of PBS. A 100 μg/mL concentration of RNase was added to the samples, which were then incubated at 37 °C for 30 min. Cells were centrifuged, resuspended in 1 mL of PBS, and stained with 10 μL PI (50 µg/mL) for 30 min at 4 °C. Data consisted of at least 2000 events analyzed by flow cytometry using the BL-3 channel for PI detection.

### 3.10. Cell Migration Assay (Scratch Assay)

Cells were seeded in a 12-well plate at a density of 80,000 cells per well. Once the cells were confluent, a vertical scratch (wound) was made from the beginning to the end of the well, and cells were immediately cultured in the presence of different concentrations of βCDPEGs, βCD, and PEGs (25, 50, 100, 250 and 500 μg/mL) that were placed on the top of the cells at a final volume of 1 mL. Then, cells were incubated for 24 h at 37 °C in a 5% CO_2_ atmosphere. Cells incubated only with culture media were taken as a control. Cell migration was monitored with an inverted microscope, and representative photographs (*n* = 4) of the cells in culture were taken immediately after the scratch (0 h) and 24 h after the treatment’s addition. Finally, the size of the scratch closure (area between the two yellow lines) for each treatment was analyzed with the Lumaview software v21.6.2 from Etaluma LS620 Microscope (San Diego, CA, USA) and plotted as the percentage of cell closure relative to the control (untreated cells). Scale bars for all images were 50 μm.

### 3.11. Statistical Analysis

We performed all of the experiments in a threefold independent manner with internal triplicates. Results were expressed as mean ± standard deviation of three independent experiments. Data were evaluated by analysis of variance (ANOVA), followed by Tukey’s multiple comparison Test, using GraphPad Prism software version 8.0.0 for Mac, (San Diego, CA, USA). The results were considered statistically significant when *p* < 0.05.

## 4. Conclusions

This work studied the cellular effects of βCDPEGs, two βCD-pegylated derivatives, through broad in vitro toxicological assays on RAW 264.7 macrophages, MC3T3-E1 osteoblasts, and MDCK cells. Aiming to understand the relationships between the pegylated molecules and specific in vitro cellular responses, we also studied the effect of the parent compounds βCD and PEGs.

βCDPEGs induced a moderate inflammatory response at high concentrations without compromising the viability of the RAW 264.7 macrophages. Although MC3T3-E1 osteoblasts were more sensitive than MDCK cells to βCDPEGs and the parent compounds, similar effects in both models were observed: the effect of βCDPEG5 on cell viability and cell cycle progression was more significant than that of βCDPEG2; in turn, PEG2 affected cell viability and cell cycle progression more than did βCDPEG2. Cell post-treatment recovery was favorable in all cases, and the compounds had similar behaviors regarding ROS overproduction. The effect on MDCK cell migration followed a comparable pattern; however, for osteoblasts, βCDPEG5 interfered with cell migration on a smaller scale than did βCDPEG2 and likewise PEG2, whose effect was shorter than its conjugate.

The present study showed that the biological response to engineered materials can be tuned through thoughtful combination of their components. In this case, the covalent conjugation of βCD and PEGs, particularly between PEG2 and βCD, resulted in an improved biocompatibility profile.

The cellular models employed in this work informed the different behavior of βCDPEGs in macrophages, osteoblasts, and epithelium-like cells, thus confirming a type of “biological library” that can provide essential information on βCDPEGs. We are currently investigating the capacity of the βCDPEG cavities to host drugs of different nature, and the obtained results, along with the biological information presented herein, will guide the potential applications of βCDPEGs.

Although βCD and PEGs have been widely studied, as far as we know, this is the first time that they have been investigated in the cellular animal models presented herein. Thus, we expect the information generated from these experiments to contribute to the rational and successful design of molecular platforms constructed from βCD and PEGs.

## Figures and Tables

**Table 1:** Global effects of βCDPEGs and PEGs on MC3T3-E1 and MDCK cells.

Cellular Response	MC3T3-E1 Osteoblasts	MDCK Cells	RAW 264.7 Macrophages
Cell viability	βCDPEG5 > βCDPEG2 PEG2 > βCDPEG2 PEG5 = βCDPEG5	βCDPEG5 > βCDPEG2 PEG2 > βCDPEG2 PEG5 = βCDPEG5	βCDPEG5 = PEG5 = βCDPEG2 = PEG2
ROS generation	PEG5 > βCDPEG5 = βCDPEG2 = PEG2	βCDPEG5 = PEG5 = βCDPEG2 = PEG2	βCDPEG5 = βCDPEG2 PEG5 = PEG2 βCDPEGs > PEGs
Cell cycle	βCDPEG5 ** > βCDPEG2 * PEG2 * > βCDPEG2 * PEG5 ** = βCDPEG5 **	βCDPEG5 * > βCDPEG2 * PEG2 * > βCDPEG2 * PEG5 * < βCDPEG5 *	N/A
^§^ Cell migration	βCDPEG5 < βCDPEG2 PEG2 < βCDPEG2 PEG5 > βCDPEG5	βCDPEG5 > βCDPEG2 PEG2 > βCDPEG2 PEG5 > βCDPEG5	N/A

* Cell cycle arrest at the S phase. ** Cell cycle arrest at the G0/G1 and S phases. ^§^ For cell migration experiments, the symbols < > express the effect on cell motility. The larger the effect, the bigger gap size and the lower % in the wound closure average.

## Data Availability

Not applicable.

## Supplementary Materials

The following supporting information can be downloaded at: https://www.mdpi.com/article/10.3390/molecules27093026/s1. Table S1: Effect of βCDPEGs, βCD, and PEGs on the cell cycle of MC3T3-E1 osteoblasts. Results are presented as mean values ± SD of triplicate experiments. Table S2: Effect of βCDPEGs, βCD, and PEGs on the cell cycle of MDCK cells. Results are presented as mean values ± SD of triplicate experiments.

## Abbreviations

αCD	α-cyclodextrin
βCD	β-cyclodextrin
HPβCD	2-Hydroxypropyl-β-cyclodextrin
IC	Inclusion complex
MβCD	Methyl-β-cyclodextrin
MW	Molecular weight
PEG	Polyethylene glycol
ROS	Reactive oxygen species
SBEβCD	Sulfobutylether-β-cyclodextrin
