# Supplementary material for: Exploring In Vitro Biological Cellular Responses of Pegylated β-Cyclodextrins
Source: Molecules. 2022 May 8;27(9):3026. doi: 10.3390/molecules27093026 (PMC9101635; doi:10.3390/molecules27093026)
Supplement: Supplementary file 1 [file molecules-27-03026-s001.zip › molecules-1714165-supplementary.pdf]

Exploring *in vitro* biological cellular responses of Pegylated β-cyclodextrins

Juliana Rincón-López[1], Miguelina Martínez-Aguilera[1], Patricia Guadarrama[1], Karla Juarez-Moreno[2,*], Yareli Rojas-Aguirre[1,*]

[1] Laboratorio de Materiales Supramoleculares (SupraMatLab), Instituto de Investigaciones en Materiales, Universidad Nacional Autónoma de México, Circuito Exterior S/N, Ciudad Universitaria, 04510, Mexico City

[2] Centro de Física Aplicada y Tecnología Avanzada, Universidad Nacional Autónoma de México, (CFATA-UNAM), Blvd. Juriquilla #3001 Col. Jurica La Mesa CP 76230, Querétaro, Qto., Mexico

*Correspondence: yareli.rojas@materiales.unam.mx (Y.R-A), kjuarez@fata.unam.mx (K. J-M)

Table S1. Effect of βCDPEGs, βCD, and PEGs on the cell cycle of MC3T3-E1 osteoblasts. Results are presented as mean values ± SD of triplicate experiments.

Compound	Parameter	Relative cell population (%)					
		control	25µg/mL	50µg/mL	100µg/mL	250µg/mL	500µg/mL
βCDPEG2	Sub G0/G1	3.7±0.1	3.7±0.4	3.7±0.2	3.8±0.4	3.4±0.3	3.8±0.3
	G0/G1	47.4±2.1	47.1±2.3	44.9±4.2	47.4±2.2	47.1±2.6	43.8±2.2
	S	18.4±0.6	16.5±4.0	15.5±3.8	15.9±0.8	18.0±5.7	23.6±3.7
	G2/M	36.1±2.3	36.4±1.4	34.0±3.5	37.4±0.8	30.6±1.6	27.9±1.3
	*Viability	100.0±9.1	97.3±3.9	100.0±1.2	76.4±14.2	85.7±4.0	77.5±9.5
βCDPEG5	Sub G0/G1	3.7±0.1	3.6±1.5	4.2±0.4	3.7±0.7	4.2±1.4	4.0±0.2
	G0/G1	47.4±2.1	45.5±2.9	54.7±4.9	56.5±2.4	57.7±1.8	56.4±1.6
	S	18.6±0.8	19.8±1.9	22.5±1.2	28.1±1.3	26.5±2.8	27.0±2.6
	G2/M	36.1±2.3	28.2±0.8	18.6±0.8	17.2±5.4	16.6±4.0	12.0±1.2
	Viability	100.0±9.1	77.4±14.9	59.5±15.2	51.1±14.4	55.3±14.4	53.9±10.4
PEG2	Sub G0/G1	3.7±0.1	2.8±.4	3.7±0.3	4.1±0.1	3.4±0.5	3.9±0.2
	G0/G1	47.4±2.1	46.4±3.4	5.2±1.0	50.2±1.5	49.7±3.5	47.7±6.9
	S	18.6±0.8	23.5±3.0	25.5±3.0	26.9±2.4	24.0±3.3	24.0±1.8
	G2/M	36.1±2.3	11.6±0.8	13.5±1.1	12.9±1.1	13.1±1.5	12.5±2.1
	*Viability	100.0±9.1	68.3±9.3	55.3±5.1	52.8±5.6	49.8±1.2	53.4±2.5
PEG5	Sub G0/G1	3.7±0.1	3.9±0.4	3.8±0.6	3.4±1.2	3.9±0.6	4.1±0.5
	G0/G1	47.4±2.1	46.8±3.4	50.5±1.4	54.9±4.0	52.4±2.7	53.7±3.2
	S	18.6±0.8	21.1±0.8	23.4±3.2	22.8±5.3	23.3±3.7	24.9±4.0
	G2/M	36.1±2.3	18.2±5.5	17.2±5.2	10.4±0.8	13.7±2.3	13.7±5.3

	*Viability	100.0±9.1	54.6±10.8	41.9±12.5	46.1±12.8	49.4±9.2	50.6±4.2
βCD	Sub G0/G1	3.7±0.1	3.9±0.1	4.0±0.1	5.5±2.1	5.2±3.2	5.9±1.6
	G0/G1	47.4±2.1	53.8±2.2	53.9±10.3	60.6±2.5	59.5±7.2	59.9±9.2
	S	18.6±0.8	21.6±8.3	24.3±3.8	24.6±3.8	21.8±2.9	18.9±5.7
	G2/M	36.1±2.3	7.8±4.0	7.8±2.4	8.8±4.8	6.4±3.5	6.3±3.5
	*Viability	100.0±9.1	77.3±10.4	54.0±3.7	59.2±8.0	37.0±28.2	70.3±4.2

*% Cell viability values obtained from the assay reported in Section 2.2.1. have been included to facilitate their comparison with the % relative cell population.

Table S2. Effect of βCDPEGs, βCD, and PEGs on the cell cycle of MDCK cells. Results are presented as mean values ± SD of triplicate experiments.

Compound	Parameter	Relative cell population (%)					
		control	25µg/mL	50µg/mL	100µg/mL	250µg/mL	500µg/mL
βCDPEG2	Sub G0/G1	4.1±0.5	3.7±0.3	3.6±0.5	3.6±1.0	3.7±0.1	3.8±0.3
	G0/G1	44.6±6.4	39.6±1.0	39.5±5.6	38.4±0.7	36.5±2.2	40.9±2.3
	S	13.0±5.4	11.9±4.3	10.7±1.1	10.9±1.7	7.2±3.3	7.4±2.5
	G2/M	31.7±0.2	28.9±1.8	30.8±1.8	27.7±1.0	29.1±5.2	30.5±3.2
	*Viability	100.0±13.0	100.0±11.7	100.0±7.2	94.8±8.1	100.0±2.5	100.0±7.7
βCDPEG5	Sub G0/G1	4.1±0.5	3.6±1.5	4.2±0.4	3.7±0.7	4.2±1.4	4.0±0.2
	G0/G1	44.6±6.4	43.1±2.5	42.8±2.8	45.8±5.9	46.5±2.2	50.2±8.0
	S	13.0±5.4	24.5±3.4	27.8±4.3	27.9±0.9	31.1±1.0	30.2±2.7
	G2/M	31.7±0.2	11.9±4.3	10.7±1.1	10.9±1.7	7.2±3.3	7.4±2.5
	*Viability	100.0±13.0	91.8±8.8	83.9±1.9	82.9±4.5	70.8±5.7	72.1±2.2
PEG2	Sub G0/G1	4.1±0.5	3.5±1.0	4.0±0.9	5.5±1.2	4.6±0.3	4.2±0.1
	G0/G1	44.6±6.4	43.6±4.1	45.2±5.5	46.8±1.1	45.0±1.9	45.3±4.3
	S	13.0±5.4	32.4±3.3	29.1±2.2	28.1±5.3	27.5±3.4	29.1±4.2
	G2/M	31.7±0.2	11.6±0.8	13.5±1.1	11.2±0.8	11.6±1.1	9.7±1.0
	*Viability	100.0±13.0	78.9±3.5	69.2±6.8	64.3±2.3	58.6±20.3	74.0±3.9
PEG5	Sub G0/G1	4.1±0.5	3.3±0.4	3.5±0.3	3.6±0.5	3.3±0.2	3.5±0.6
	G0/G1	44.6±6.4	45.4±3.1	47.9±10.4	56.3±1.2	47.3±7.4	46.4±7.6
	S	13.0±5.4	26.0±3.4	28.9±1.4	25.0±0.9	26.8±2.9	25.4±2.8
	G2/M	31.7±0.2	13.1±2.3	11.5±1.7	11.7±0.6	11.6±1.1	16.7±3.4

	*Viability	100.0±13.0	79.5±5.4	76.9±2.5	71.8±7.1	71.1±10.7	81.4±5.6
βCD	Sub G0/G1	4.1±0.5	2.8±0.9	3.7±0.3	4.9±1.1	4.1±1.1	3.5±0.7
	G0/G1	44.6±6.4	42.0±8.9	36.0±7.4	41.8±3.7	41.8±3.2	45.1±5.0
	S	13.0±5.4	13.3±2.2	38.6±2.4	35.2±1.2	35.6±3.1	37.8±4.0
	G2/M	31.7±0.2	37.8±12.4	17.1±4.1	9.4±1.3	2.7±0.5	7.2±2.6
	*Viability	100.0±13.0	95.1±5.8	97.6±9.4	73.7±6.4	79.0±2.7	76.1±2.8

*% Cell viability values obtained from the assay reported in Section 2.3.1. have been included to facilitate their comparison with the % relative cell population.
